# Supplementary material for: Identification of osteoclast-osteoblast coupling factors in humans reveals links between bone and energy metabolism
Source: Nat Commun. 2020 Jan 7;11:87. doi: 10.1038/s41467-019-14003-6 (PMC6946812; doi:10.1038/s41467-019-14003-6)
Supplement: Supplementary file 2 — Supplementary Information [file 41467_2019_14003_MOESM2_ESM.pdf]

Supplementary Figure 1

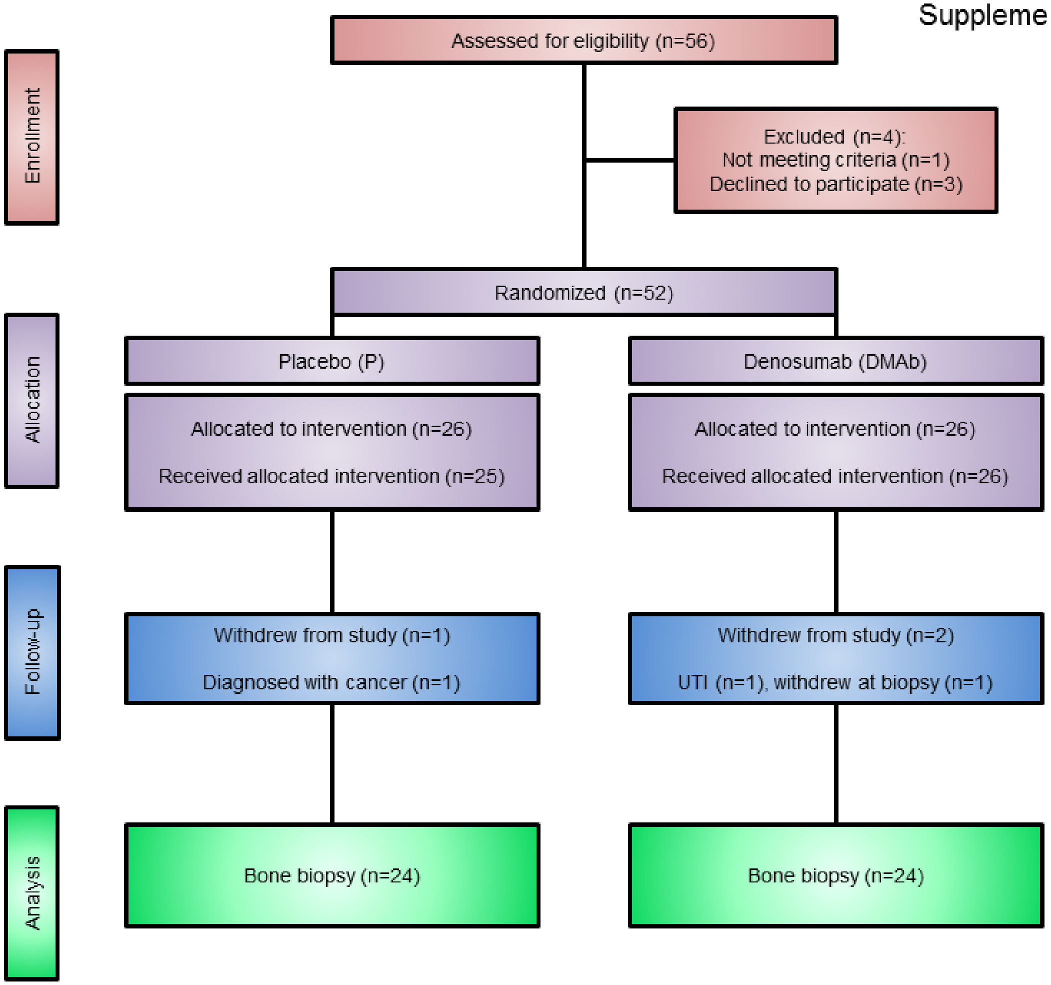

Supplementary Figure 1. CONSORT flow diagram for the interventional study. UTI, urinary tract infection.

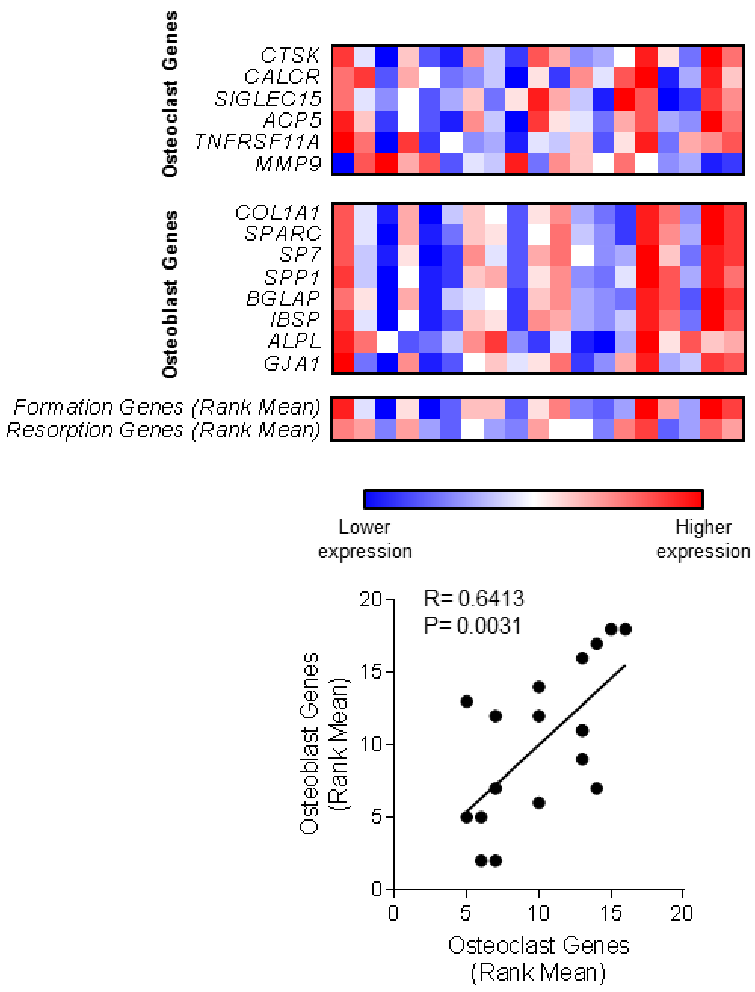

Supplementary Figure 2. DMAb-suppressed osteoclast and osteoblast genes correlate in an independent whole bone RNA-sequencing dataset. Normalized RNA-sequencing values (CQN) were ranked per gene. (A) The heat map compares individual subject ranks per gene with red denoting higher expression and blue lower expression; (B) Rank mean values for osteoclast and osteoblast gene sets were plotted against each other to show correlation between the gene sets in whole bone sequencing data (N=19 participant biopsies). The strength of the correlation was determined using Spearman's rank correlation. Source data are provided as a Source Data file.

Supplementary Figure 3

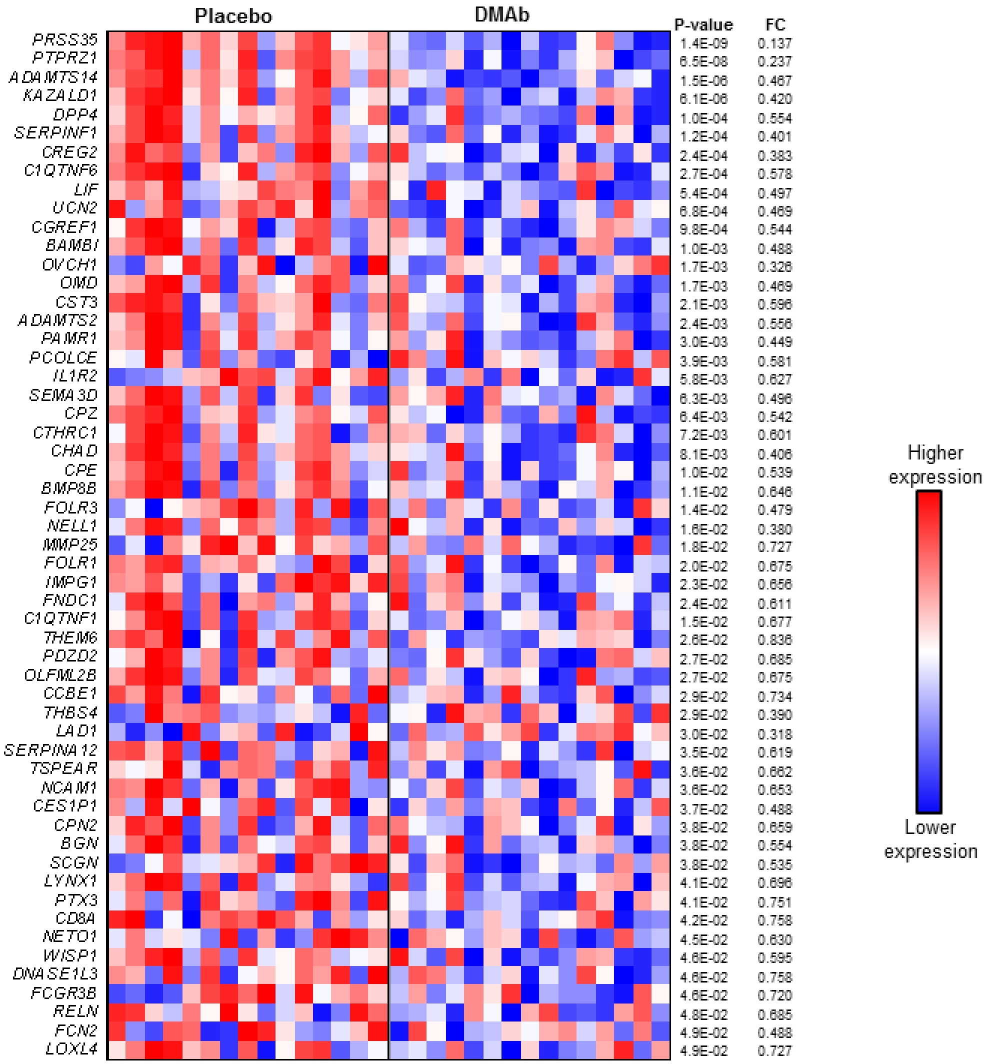

Supplementary Figure 3. Secreted genes significantly suppressed by DMAb treatment. Normalized RNA-seq values (CQN) were ranked for each gene. The heat map compares individual subject ranks per gene with red denoting higher expression and blue lower expression. The p-value reflects significance between the Placebo and DMAb subjects, and the fold chaing (FC) reflects fold change of DMAb as compared to Placebo (N=15 participant biopsies/group). P-values and FC were calculated using the R program edgeR. Source data are provided as a Source Data file.

Supplementary Figure 4

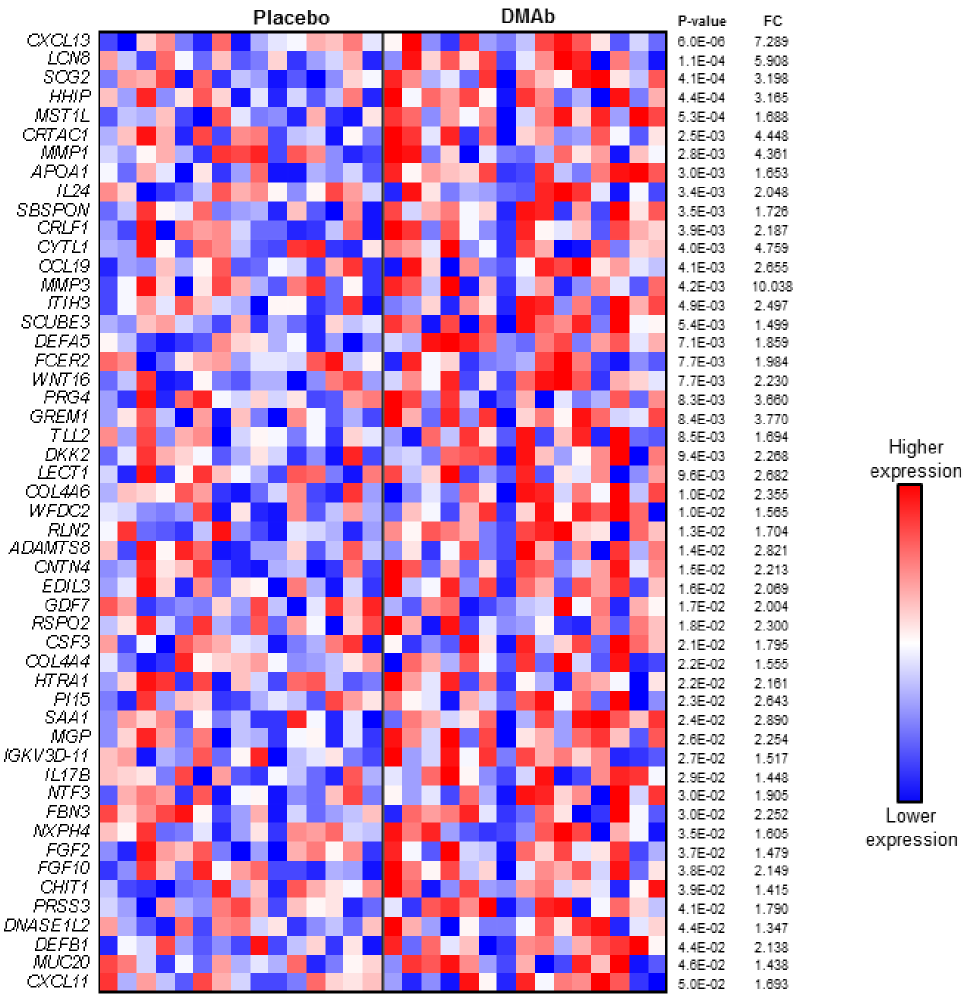

Supplementary Figure 4. Secreted genes significantly upregulated by DMAb treatment. Normalized RNA-seq values (CQN) were ranked for each gene. The heat map compares individual subject ranks per gene with red denoting higher expression and blue lower expression. The p-value reflects significance between the Placebo and DMAb subjects, and the FC reflects fold change of DMAb as compared to Placebo (N=15 participant biopsies/group). P-values and FC were calculated using the R program edgeR. Source data are provided as a Source Data file.

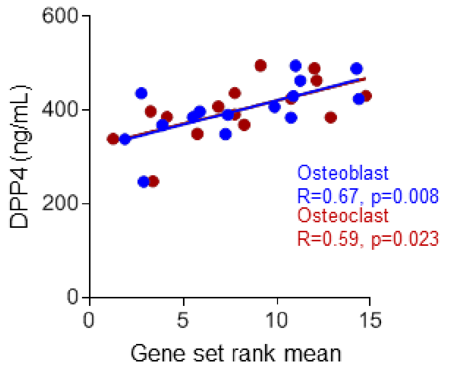

Supplementary Figure 5. Correlation of circulating DPP4 levels with gene sets. Peripheral serum DPP4 levels correlate with osteoblast and osteoclast gene sets in the placebo participants; Spearman's correlation coefficient was used to determine strength of relationships. N=15 participant samples per group. Source data are provided as a Source Data file.

Supplementary Tables

Supplementary Table 1. Gene ontology analysis of differentially expressed genes in the DMAb bone biopsies. P-values to assess Biological Processes affected by DMAb were calculated using Ingenuity Pathway Analysis. N=15 participant biopsies per group. Source data are provided as a Source Data file.

Gene Ontology Biological Processes	# Genes Differentially Expressed in DMAb bone biopsies	P-value
Ossification	34 (364)	5.90E-07
Bone Development	21 (218)	3.30E-05
Bone Remodeling	10 (97)	1.80E-04
Osteoblast Differentiation	16 (188)	0.001
Bone Mineralization	12 (108)	0.002
Bone Trabecula Formation	3 (12)	0.004
Bone Morphogenesis	8 (114)	0.006
Osteoclast Differentiation	8 (105)	0.006
Bone Resorption	7 (72)	0.008
**Total Bone Related Genes**	**48 (555)**	

Supplementary Table 2. Gene ontology database bone-related biological processes genes significantly altered in DMAb bone biopsies. Genes that are known osteoclast markers are highlighted in red and genes that are known osteoblast lineage markers are in blue. P-value reflects significance between the DMAb and placebo treated subjects. FC reflects fold change in DMAb relative to Placebo biopsies (R package edgeR). N=15 participant biopsies/group. Source data are provided as a Source Data file.

Gene	Biological Processes	P-value	FC
COL1A1	Bone Development, Bone Morphogenesis, Bone Trabecular Formation, Ossification	1.77E-08	0.13
TNN	Ossification, Osteoblast Differentiation	1.19E-08	0.16
MMP13	Bone Development, Bone Mineralization, Bone Morphogenesis	1.54E-05	0.20
CTSK	Bone, Remodeling, Bone Resorption	5.33E-08	0.20
CALCR	Bone Remodeling, Bone Resorption, Osteoclast Differentiation	3.87E-08	0.22
SIGLEC15	Bone Development, Bone Remodeling, Bone Resorption, Osteoclast Differentiation	2.02E-12	0.22
CREB3L1	Ossification, Osteoblast Differentiation	4.52E-08	0.22
SPARC	Bone Development	1.72E-06	0.23
ACP5	Bone Development, Bone Morphogenesis, Bone Remodeling, Bone Resorption, Ossification	5.63E-10	0.24
DCSTAMP	Bone Remodeling, Bone Resorption, Osteoclast Differentiation	0.0007	0.33
IFITM5	Bone Morphogenesis, Ossification	1.3E-05	0.33
OCSTAMP	Osteoclast Differentiation	0.0004	0.36
NELL1	Bone Mineralization, Osteoblast Differentiation	0.016	0.38
CHAD	Bone Development, Bone Trabecula Formation	0.008	0.41
SP7	Ossification, Osteoblast Differentiation	0.0002	0.41
KAZALD1	Ossification	6.12E-06	0.42
SPP1	Bone Remodeling, Bone Resorption, Ossification, Osteoblast Differentiation	0.005	0.42
BGLAP	Bone Development, Bone Mineralization, Ossification, Osteoblast Differentiation	0.002	0.45
OMD	Bone Mineralization, Ossification	0.002	0.47
IBSP	Bone Mineralization, Ossification	0.027	0.47
COL13A1	Ossification	0.001	0.49
MMP16	Bone Development, Bone Morphogenesis, Ossification	0.001	0.50
TNFRSF11A	Bone Remodeling, Bone Resorption, Ossification, Osteoclast Differentiation	0.0007	0.54
SLC9B2	Bone Development, Osteoclast Differentiation	1.12E-05	0.56
SATB2	Ossification, Osteoblast Differentiation	0.001	0.56
CADM1	Bone Development	0.005	0.57
TMEM119	Bone Development, Bone Mineralization, Ossification, Osteoblast Differentiation	0.009	0.58
CTHRC1	Ossification, Osteoblast Differentiation	0.007	0.60
ALPL	Bone Development, Bone Morphogenesis, Ossification	0.001	0.61
MMP9	Ossification	0.003	0.63
LRP4	Ossification	0.027	0.63
GJA1	Bone Mineralization, Bone Remodeling, Ossification, Osteoblast Differentiation	0.025	0.63
BMP8B	Ossification	0.011	0.65
HOXA2	Ossification, Osteoblast Differentiation	0.004	0.68
SH3PXD2B	Bone Development, Ossification, Osteoblast Differentiation	0.025	0.69
SERPINH1	Bone Development, Bone Morphogenesis	0.028	0.71
GPR68	Bone Development, Osteoclast Differentiation	0.016	0.76

SH3PXD2A	Osteoclast Differentiation	0.024	0.77
EXT1	Ossification	0.049	0.77
SPNS2	Bone Development	0.049	0.86
BCL2	Ossification	0.049	1.23
FGF2	Ossification, Osteoblast Differentiation	0.037	1.48
WNT16	Bone Remodeling	0.008	2.23
MGP	Bone Mineralization, Ossification	0.026	2.25
RSPO2	Bone Mineralization, Ossification, Osteoblast Differentiation	0.018	2.30
HTR1B	Bone Remodeling	4.73E-05	3.30
GREM1	Bone Mineralization, Bone Remodeling, Bone Trabecula Formation, Ossification, Osteoblast Differentiation	0.008	3.77
ACTN3	Bone Development, Bone Mineralization, Bone Morphogenesis, Ossification, Osteoblast Differentiation	0.002	7.02

Supplementary Table 3. Correlation of DMAb-suppressed osteoblast and osteoclast genes to (A) circulating markers of bone formation (PINP, OCN) and bone resorption (CTX, TRAP5b) and (B) micro-CT parameters from the bone biopsies, both in the placebo-treated subjects. * denotes $p<0.05$, N=15 participant biopsies; Spearman rank correlation. Source data are provided as a Source Data file.

A

	**Osteoblast gene set**		**Osteoclast gene set**	
	**R**	**P-value**	**R**	**P-value**
**P1NP**	0.55	0.035*	0.32	0.249
**OCN**	0.18	0.532	-0.18	0.517
**CTX**	0.57	0.029*	0.44	0.102
**TRAP5b**	0.06	0.839	-0.08	0.784

B

	**Osteoblast gene set**		**Osteoclast gene set**	
	**R**	**P-value**	**R**	**P-value**
**BV/TV**	**0.08**	**0.783**	**-0.36**	**0.190**
**Conn.Dens**	**-0.25**	**0.361**	**-0.57**	**0.029***
**SMI**	**0.15**	**0.593**	**0.13**	**0.646**
**Tb.N**	**-0.38**	**0.161**	**-0.66**	**0.009***
**Tb.Th**	**0.19**	**0.498**	**-0.2055**	**0.460**
**Tb.Sp**	**0.40**	**0.145**	**0.66**	**0.010***

Supplementary Table 4. Olinks summary results and correlation to osteoblast and osteoclast gene sets. (A) Proteins significantly altered in DMAb-treated participant bone marrow plasma, as assessed by Olinks immuno-PCR based assay. P-value reflects difference between DMAb and Placebo treated subjects (N=24 participant samples/group; Kruskal Wallis). Proteins highlighted in red signify proteins that were also regulated at the RNA gene expression level according to RNA-sequencing results. (B) Placebo bone marrow plasma protein levels correlating to placebo osteoblast or osteoclast rank gene sets (N=15 participant samples). Correlations determined using Spearman rank correlation. Source data are provided as a Source Data file.

A) Placebo vs DMAb BM plasma protein

Protein Assay	P-Value	FC
NRP1	0.021988	-1.05824
COL1A1	1.07E-18	-0.47267
IL-17A	0.041643	-0.46361
VASN	0.034997	-0.30089
OPN	2.54E-09	-0.19352
MEPE	0.020308	-0.1683
GDNF	0.044836	-0.16039
PAM	0.017655	-0.14742
CD59	0.01008	-0.14323
FCGR3B	0.025378	-0.141
LAMP3	0.004703	-0.13817
TNFRSF9	0.000364	-0.11773
EFEMP1	0.038596	-0.11572
FAP	0.019874	-0.09591
TNFRSF9	0.00175	-0.09487
DLK-1	0.042208	-0.0905
ITGAM	0.02013	-0.08956
TNFRSF10C	0.016196	-0.08637
CDH1	0.024998	-0.07945
COL18A1	0.046301	-0.07935
IGFBP6	0.018282	-0.07655
TGFBR3	0.019356	-0.07443
CD46	0.035197	-0.07291
TNFRSF11A	0.040678	-0.0684
PDCD1	0.048463	-0.0679
CCL14	0.030839	-0.0663
CXCL10	0.031762	-0.05919
TIMP1	0.015029	-0.05898
IGLC2	0.038827	-0.05821
TNFRSF12A	0.006554	-0.05793
CX3CL1	0.044689	-0.05624
CST3	0.033728	-0.04492
NOTCH1	0.046424	-0.04324
VEGFA	0.036997	-0.04311
GAS6	0.032267	-0.04129
U-PAR	0.031584	-0.03429
CCL4	0.04554	-0.03401
IL-10RB	0.044888	-0.03329
CCL23	0.004971	-0.03278
AXL	0.027405	-0.03134
CCL23	0.026141	-0.02977
CD93	0.010975	-0.02543
PGF	0.032143	-0.02182
CSF-1	0.044242	-0.01679
PIgR	0.037558	-0.01587
SERPINA12	0.019254	0.156792
TRANCE	8.31E-09	0.22393
PTPRS	0.008031	0.71461

B) Placebo BM Plasma Protein correlations with osteoblast or osteoclast gene sets

Protein Assay	P-value	R (Osteoblasts)
DPP4	0.0022	0.7429
ITGAM	0.0811	0.4667
TNXB	0.0834	0.4643
IL12	0.0915	0.4536
CX3CL1	0.0972	-0.4464
NRP1	0.0944	-0.45
CSF-1	0.0915	-0.4536
MMP12	0.0834	-0.4643
MCP-4	0.0834	-0.4643
CXCL9	0.0808	-0.4679
MB	0.0759	-0.475
IGFBP-2	0.0688	-0.4857
CD40-L	0.0688	-0.4857
CXCL9	0.0688	-0.4857
NT-proBNP	0.0666	-0.4893
CCL17	0.0644	-0.4929
MMP12	0.0562	-0.5071
FGF-23	0.0562	-0.5071
IL10	0.0524	-0.5143
GDF-15	0.0362	-0.55
TFF3	0.0195	-0.6036
FGF-23	0.0089	-0.6607

Protein Assay	P-value	R (Osteoclasts)
PLA2G7	0.0102	0.6506
DPP4	0.0179	0.6095
FCN2	0.0527	0.513
PON3	0.0812	0.4665
AGRP	0.0838	0.4629
OPG	0.0977	-0.445
PD-L1	0.092	-0.4522
TNFRSF11A	0.0892	-0.4558
NCR1	0.0892	-0.4558
REG3A	0.0881	-0.4569
MCP-4	0.0877	-0.4576
CCL23	0.0865	-0.4593
IL-1RT1	0.0838	-0.4629
TWEAK	0.0824	-0.4647
TNFRSF14	0.0812	-0.4665
LTBR	0.0725	-0.479
CA1	0.0679	-0.4861
CD40-L	0.0626	-0.4951
EPHB4	0.0605	-0.4987
TNF-R1	0.0565	-0.5058
MMP12	0.0554	-0.5076
TNF-R2	0.049	-0.5201
CXCL9	0.049	-0.5201
TFF3	0.0424	-0.5344
ADM	0.0415	-0.5362
IL6	0.0385	-0.5433
DNER	0.0385	-0.5433
PGF	0.0378	-0.5451
CX3CL1	0.0378	-0.5451
MMP12	0.0324	-0.5594
PRELP	0.0299	-0.5666
CXCL9	0.0299	-0.5666
MB	0.0293	-0.5684
ANGPT2	0.0293	-0.5684
CD40	0.0275	-0.5737
IL10	0.0253	-0.5809
CCL23	0.0253	-0.5809
CA3	0.0243	-0.5845
CSF-1	0.0116	-0.6416
IgG Fc receptor II-b	0.0108	-0.647
CSF-1	0.0026	-0.7328

Supplementary Table 5. Percent change in serum metabolic markers in placebo and DMAb treated participants. Data are expressed as percent change compared to baseline with standard deviation. Mann-Whitney was used to determine significance. N=24 placebo, N=22 DMAb participant samples. Source data are provided as a Source Data file.

**(% Change)**	**Placebo (N=24)**	**DMAb (N=22)**	**p-value**
GIP	4.71 (34.05)	-2.05 (38.84)	0.623
Total Cholesterol	-18.29 (19.03)	-17.18 (23.49)	0.422
LDL Cholesterol	-10.79 (14.44)	-12.10 (19.64)	0.711
HDL cholesterol	-4.62 (5.40)	-4.55 (6.30)	0.601
Triglycerides	-14.38 (44.71)	-2.68 (27.43)	0.524
HOMA-IR	-3.18 (20.47)	-5.91 (8.13)	0.149
HOMA-β	-0.12 (0.74)	-0.33 (0.35)	0.054

Supplementary Table 6. Baseline clinical characteristics of the subjects who were treated with calcium/vitamin D alone, a bisphosphonate, or DMAb (ANCOVA and Bonferroni). N=115 patients per group. Source data are provided as a Source Data file.

	Treatment			
**Characteristic**	**Calcium/Vitamin D N=115**	**Bisphosphate n=115**	**DMAb n=115**	**p-value**
**Gender, n (%)**				0.069
**Female**	79 (68.7)	76 (66.1)	91 (79.1)	
**Male**	36 (31.1)	39 (33.9)	24 (20.9)	
**Age, yrs**				0.010
**Mean (SD)**	71.4 (9.5)	70.3 (9.6)	74.1 (10.0)	
**Range**	46.0-90.0	45.0-91.0	46.0-93.0	
**BMI, kg/m^2**				0.126
**Mean (SD)**	31.9 (7.7)	30.8 (5.9)	29.9 (8.6)	
**Range**	18.7-60.6	15.1-46.7	17.9-70.8	
**Diabetes/prediabetes duration, yrs**				0.091
**Mean (SD)**	11.5 (9.4)	10.4 (9.7)	13.4 (12.3)	
**Range**	1.0-43.0	1.0-52.0	1.0-69.0	
**HbA1c n6m, %**				0.154
**Mean (SD)**	7.0 (1.1)	7.0 (1.4)	6.7 (1.2)	
**Range**	4.6-11.7	5.0-18.9	5.1-12.3	
**FPG, mg/dL**				0.569
**N-Miss**	15	27	6	
**Mean (SD)**	141.8 (60.0)	141.3 (44.1)	135.0 (48.3)	
**Range**	52.0-480.0	81.0-348.0	64.0-301.0	
**Diabetes treatment, n (%)**				< 0.001
**Lifestyle**	26 (22.6)	26 (22.6)	47 (40.9)	
**Insulin/Insulin+other**	38 (33.0)	37 (32.2)	44 (38.3)	
**Other**	51 (44.4)	52 (45.2)	24 (20.9)	

Supplementary Table 7. Change in HbA1c and FPG in diabetic or pre-diabetes patients treated with Calcium/VitD, Bisphosphonate, or DMAb for one year adjusting for baseline covariates and change in BMI. Because we found that DMAb patients exhibited decreased BMI, we tested whether the effects of DMAb to reduce HbA1c may be secondary to reduced BMI. After adjusting for change in BMI, these findings reveal that there is still a significant reduction in HbA1c in DMAb patients. Data are presented as predicted marginal mean values with standard deviation (ANCOVA and Bonferroni). N patients per group is noted for each individual group. Source data are provided as a Source Data file.

			Treatment			Treatment	DMAb vs Ca/VitD	DMAb vs Bisphoshonate
Outcomes	Time point	N	Calcium/Vitamin D	Bisphosphate	Denosumab	p-value	p-value	p-value
Change in HbA1c (%)	0-6 mo	256	0.052 (0.110)	0.227 (0.111)	-0.131 (0.110)	0.083	0.245	0.026
	0-12 mo	318	0.126 (0.096)	0.356 (0.096)	-0.239 (0.109)	< 0.001	0.013	< 0.001
Change in FPG (mg/dL)	0-6 mo	215	4.840 (5.000)	8.014 (5.349)	-7.909 (4.533)	0.059	0.064	0.028
	0-12 mo	256	0.998 (3.804)	-0.922 (4.227)	-8.827 (4.064)	0.199	0.081	0.194

Supplementary Table 8. PCR primers used in the study.

Gene Symbol	Accession #	Forward Primer Sequence (5' to 3')	Reverse Primer Sequence (5' to 3')
ACTB	NM_001101	CCCAGCCATGTACGTTGCTAT	TCACCGGAGTCCATCACGAT
ADAMTS14	NM_080722	GAGGGAACACGATTTCTCAG	GCCAGGTAGAAGATGAAGATG
ADAMTS2	NM_021599	CCGGGACTGCACTACTCCAT	AACGCCGTGCACATCATGTA
B2M	NM_004048	GCCGTGTGAACCATGTGACTT	CAAACCTCCATGATGCTGCTT
BAMBI	NM_012342	CAGACCTCAGCAACGATAAG	TAGTTCAGATAAGACTCCGTCA
BGN	NM_001711	GATCAGGATGATCGAGAACG	CCAACTTGTTGTTGTCCAAG
BMP8B	NM_001720	GTCCTATCCATGCCCCTTACTG	AGGTGCCTCTCAGGTCATTCC
C1QTNF1	NM_030968	GGAAACTACCTCTGGCTTAAT	CCACCTATCCCTCAAGAAAG
C1QTNF6	NM_031910	CGTGGCATCTACTTCTTCAG	ACAGCCTCTTTCTGGTTATG
CCBE1	NM_133459	TCTCTCCACTCCTCTTCTTC	CATTCACTCCCTCATGTCTG
CD8A	NM_001145873	GAGAGAACGAGGGCTACTAT	GGCTGAAGTACATGATGGAG
CGREF1	NM_001301324	CCAGGAAGGATGTTACCTTTG	CCTTGTGACTCCATCCTTTG
CHAD	NM_001267	CAAGTCAATCAGAACCACAAC	GGAAAGAGGAACAGAGGAAG
CPE	NM_001873	GACCTACTGGGAGGATAACA	TTGAAGGTCTCGGACAAATC
CPN2	NM_001080513	CCTACACCCTGAGGTCTATT	TTTGAGCCAGATGTCAGAAG
CPZ	NM_001014447	AATAGGTGTGGAGGCAATTT	CAGGTAGTTGAAATCGGACA
CREG2	NM_153836	CCTGATGGAGTGAGAAGAAAG	AACAAAGAGACAGTGGTCAATA
CST3	NM_001288614	AGGAAAGCATTCTGCTCTTT	GTGGATTTCGACAAGGTCAT
CTHRC1	NM_138455	TGCAGAAATGCATGCTGTCA	GGGAAGAGGTCCTGAACATTCA
DNASE1L3	NM_001256560	GCTTACAAGCTGACTGAAGA	GCCCTTGAAGACTGTAGTTTA
DPP4	NM_001935	GACTGGTTCAAATGTTGTTCTC	TCTCTCCCTAATCCCTCTTATC
FCGR3B	NM_001329120	GACTGTGAACATCACCATCA	TACCATCACCAAGCAGAAAG
FCN2	NM_015837	GGACTTTGAGGACAACTACC	CCAGGACCAGATTGTACTTC
FNDC1	NM_032532	CAAACATGATCCCAGCTACA	CTCCCACGAACTTTCGATAC
FOLR1	NM_000802	TGCCAACCTTTCCATTTCTA	GTAGTTGCTGACCTTGTAGG
FOLR3	NM_000804	GGGATCACTGTGGTAAGATG	GTGAGCACTCATAGAGACAG
G6PD	NM_000402	TGACATCCGCAAACAGAGTGA	GCAAAGAAGTCCTCCAGCTTGA
GAPDH	NM_002046	GACCTGACCTGCCGTCTAGAAA	CCTGCTTCACCACCTTCTTGA
GUSB	NM_000181	TGGTTGGAGAGCTCATTTGGA	ACTCTCGTCGGTGACTGTTCAG
IL1R2	NM_004633	GGCACACCCTTAACCACCAT	GCGCTCTCTATGTGGGTGTCA
IMPG1	NM_001563	CACTCCTGTCTCAGCTTTAC	GCAGACTGAAGAACACTACC
KAZALD1	NM_001319303	CTTAGAACTAGTGGGAAGGC	CACGAACTTGGATTCAGAAAG
LAD1	NM_005558	CTCGGAAACAACCCTAACTC	GGTATCTCTCCAGCTTCTCT
LIF	NM_002309	TCTGAAAACTGCCGGCATCT	ACCTTCATTATGGGCTGCACTT
LOXL4	NM_032211	GTGGGTGGATATCACAGATG	CTGACTCTGCCACTTCATAG
LYNX1	NM_177477	CCAGGATGAAGGTCAGTAAG	GCTTGGAGTAGCCATCATAC
MMP25	NM_022468	GATTGCCTCTGGAACTATTCT	TTCCCAGTGATTCTGATGTG
NCAM1	NM_000615	GGGCATCCTCATCGTCATCTT	GCACATGAACAGGCCACACTT
NELL1	NM_001288714	TTTGTCCCATCTACTGCATAC	GTATAATGCGACTGGACTCTAC
NETO1	NM_001201465	TGCAGAGGGAGGTATCTTTA	GAGCGGCTTCTATGATGTAG
OLFML2B	NM_001347700	TGGAGGAGGAAGTGTCTAAA	TTTGCCTCGCTTATTCATCT
OMD	NM_005014	TCACGATGATCCTGACAATGC	CAAAGTGCCCTTCTGCTCCTT
OVCH1	NM_001353179	TAGAAGAGGTGGTGGAATCT	CCTTCACATGGTTGTTTCTTAC
PAMR1	NM_015430	AAATTCTACCGGGATGATGAC	AGTTGGGATGCAGAATGATAG
PCOLCE	NM_002593	TCCTCCGAAGGGAATGAACTC	GTAGGAGGCTGAGAAGCCATCA

PDZD2	NM_178140	GAGCCAAGAGTTGGATTAGG	GTGAATGTAGATGCCAGGAG
POLR2A	NM_000937	GCCACCCAGATGACCTTGAATA	CACCCAGCGTCACATTCTTG
PRSS35	NM_001170423	GGCTAACAGAGACCTGAAAC	GGCATAACCTATGAAGTGATCT
PTPRZ1	NM_001206838	CCGAGAAGAAGGCAGTTATAC	AGATGAGAATACCCACAAGAAC
PTX3	NM_002852	GCTGTATCTCAGCTACCAATC	CAGTTTGTTCTCCTCTCCAC
RELN	NM_173054	GAGACTGGGATGTGGTAAAG	CCACCGTTGAGAGAATACTG
RPL13A	NM_012423	TGAGTGAAAGGGAGCCAGAAG	CAGATGCCCCACTCACAAGAT
SCGN	NM_006998	TGAGGCTAAACTGGAAGAATAC	GATCCAACCGACCATCTTTAT
SEMA3D	NM_152754	GACCACCACTACATCAGAAC	GGATTGTAGGTGTCTGGTATG
SERPINA12	NM_173850	GGCTATGACGATAAGCTCTC	GGATGAAGATGGCTGTGATA
SERPINF1	NM_002615	CGCTGGACTATCACCTTAAC	GAATCTTGCCAATGAAGAGAAG
SPHK1	NM_021972	TGGCAGCTTCCTTGAACCAT	TGGTCAGGAGGTCTTCATTGG
TBP	NM_003194	GGTTTGCTGCGGTAATCATGA	CTCCTGTGCACACCATTTTCC
THBS4	NM_003248	GTTTTGCCAAGTCAAACAAGCA	GCACGCTCCATTTCGACACT
THEM6	NM_001363000	CCTCTATATTCAGCATGTTCCT	CATTAGGGAGACCTGAGAAG
TSPEAR	NM_144991	GTCATCAACTCCGTCATCTAC	TGAGAATGTCCTGGAACTTG
TUBA1A	NM_006009	GAGTGCATCTCCATCCACGTT	TAGAGCTCCCAGCAGGCATT
UCN2	NM_033199	CACTGTCATGCAGGTCATATC	AGTGACCCAACTTAGCAATG
WISP1	NM_003882	CCGCCAGGTCCTATGGATTAA	GCAAAGATGTCATTGGGATTCC
