# Supplementary material for: Identification of osteoclast-osteoblast coupling factors in humans reveals links between bone and energy metabolism
Source: Nat Commun. 2020 Jan 7;11:87. doi: 10.1038/s41467-019-14003-6 (PMC6946812; doi:10.1038/s41467-019-14003-6)
Supplement: Supplementary file 3 — Description of Additional Supplementary Files [file 41467_2019_14003_MOESM3_ESM.pdf]

Supplementary data 1: Olink proteomic datasets

Description: The data files for the Olink proteomic analysis
