# Supplementary material for: Identification of osteoclast-osteoblast coupling factors in humans reveals links between bone and energy metabolism
Source: Nat Commun. 2020 Jan 7;11:87. doi: 10.1038/s41467-019-14003-6 (PMC6946812; doi:10.1038/s41467-019-14003-6)
Supplement: Supplementary file 4 — Reporting Summary [file 41467_2019_14003_MOESM4_ESM.pdf]

nature research

Corresponding author(s): Khosla, Ourler

Last updated by author(s): Nov 6, 2019

# Reporting Summary

Nature Research wishes to improve the reproducibility of the work that we publish. This form provides structure for consistency and transparency in reporting. For further information on Nature Research policies, see Authors & Referees and the Editorial Policy Checklist.

## Statistics

For all statistical analyses, confirm that the following items are present in the figure legend, table legend, main text, or Methods section.

| n/a | Confirmed | |
|---|---|---|
| ☐ | ☒ | The exact sample size (*n*) for each experimental group/condition, given as a discrete number and unit of measurement |
| ☐ | ☒ | A statement on whether measurements were taken from distinct samples or whether the same sample was measured repeatedly |
| ☐ | ☒ | The statistical test(s) used AND whether they are one- or two-sided<br>*Only common tests should be described solely by name; describe more complex techniques in the Methods section.* |
| ☐ | ☒ | A description of all covariates tested |
| ☐ | ☒ | A description of any assumptions or corrections, such as tests of normality and adjustment for multiple comparisons |
| ☐ | ☒ | A full description of the statistical parameters including central tendency (e.g. means) or other basic estimates (e.g. regression coefficient) AND variation (e.g. standard deviation) or associated estimates of uncertainty (e.g. confidence intervals) |
| ☐ | ☒ | For null hypothesis testing, the test statistic (e.g. *F*, *t*, *r*) with confidence intervals, effect sizes, degrees of freedom and *P* value noted<br>*Give P values as exact values whenever suitable.* |
| ☒ | ☐ | For Bayesian analysis, information on the choice of priors and Markov chain Monte Carlo settings |
| ☒ | ☐ | For hierarchical and complex designs, identification of the appropriate level for tests and full reporting of outcomes |
| ☐ | ☒ | Estimates of effect sizes (e.g. Cohen's *d*, Pearson's *r*), indicating how they were calculated |

*Our web collection on statistics for biologists contains articles on many of the points above.*

## Software and code

Policy information about availability of computer code

| | |
|---|---|
| Data collection | The RNA sequencing data was processed using Mayo Clinic's MAP-Rseq (v2.1.0) application. The R package edgeR51 was used to identify which genes were differentially expressed between the Placebo and DMAb treated subjects. |
| Data analysis | Statistical analyses for all analyses were conducted using SAS (version 9.4; Cary, NC) and R (version 3.4.2; Vienna, Austria). Ingenuity Pathway Analysis (IPA) Software was used to identify secreted genes significantly altered by denosumab treatment. Data were plotted using GraphPad Prism Software. |

For manuscripts utilizing custom algorithms or software that are central to the research but not yet described in published literature, software must be made available to editors/reviewers. We strongly encourage code deposition in a community repository (e.g. GitHub). See the Nature Research guidelines for submitting code & software for further information.

## Data

Policy information about availability of data

All manuscripts must include a data availability statement. This statement should provide the following information, where applicable:

- Accession codes, unique identifiers, or web links for publicly available datasets
- A list of figures that have associated raw data
- A description of any restrictions on data availability

The data that support the findings of this study are available from the corresponding author upon reasonable request.





# Field-specific reporting

Please select the one below that is the best fit for your research. If you are not sure, read the appropriate sections before making your selection.

☒ Life sciences ☐ Behavioural & social sciences ☐ Ecological, evolutionary & environmental sciences

For a reference copy of the document with all sections, see nature.com/documents/nr-reporting-summary-flat.pdf

# Life sciences study design

All studies must disclose on these points even when the disclosure is negative.

| | |
|---|---|
| Sample size | Sample size (N=25 for placebo vs denosumab) was determined based on a previous clinical study of the effects of estrogen on human bone needle biopsy gene expression. For the study of diabetes and pre-diabetes patients, all subjects were identified with the i2b2 search engine in the Mayo Clinic patient clinical data base, and their medical records were reviewed; total sample size is based on eligible patients. Subjects were excluded if baseline, or 6 or 12 month HbA1c, FPG and weight, and duration of type 2 diabetes mellitus or prediabetes were not available. A total of 253 potential subjects with type 2 diabetes mellitus or prediabetes treated with DMab for osteoporosis were identified. Of these, 138 were excluded due to failure to meet inclusion criteria, and 115 were eligible for inclusion in the DMab-treated group. One hundred and fifteen subjects treated with a bisphosphonate (either oral; n=83 or intravenous; n=32) and 115 subjects treated with calcium and vitamin D who matched the DMab subjects for age, sex, BMI, and duration of type 2 diabetes or prediabetes also were identified. |
| Data exclusions | Two DMAb values from Study A were excluded from secondary analysis of metabolic parameters (glucose, insulin, GLP1) because of failure to fast for baseline serum measurements (N=1) and Type 2 Diabetes (N=1). |
| Replication | Correlations of bone formation and resorption gene sets identified in the current study were assessed in a previous bone biopsy dataset to confirm the validity of these correlations. |
| Randomization | For the first study comparing denosumab vs placebo bone biopsy gene expression (Study A)- following recruitment, patients were randomly assigned to placebo vs denosumab. |
| Blinding | Investigators were blinded to treatment during treatment and sample collection/cell isolation, as well as during assessment of serum markers. |

# Reporting for specific materials, systems and methods

We require information from authors about some types of materials, experimental systems and methods used in many studies. Here, indicate whether each material, system or method listed is relevant to your study. If you are not sure if a list item applies to your research, read the appropriate section before selecting a response.

### Materials & experimental systems

| n/a | Involved in the study | |
|---|---|---|
| ☒ | ☐ | Antibodies |
| ☒ | ☐ | Eukaryotic cell lines |
| ☒ | ☐ | Palaeontology |
| ☒ | ☐ | Animals and other organisms |
| ☐ | ☒ | Human research participants |
| ☐ | ☒ | Clinical data |

### Methods

| n/a | Involved in the study | |
|---|---|---|
| ☒ | ☐ | ChIP-seq |
| ☒ | ☐ | Flow cytometry |
| ☒ | ☐ | MRI-based neuroimaging |

## Human research participants

Policy information about studies involving human research participants

| | |
|---|---|
| Population characteristics | For the first study, all women were aged 50–80 years old with a BMI between 18 and 34 kg/m2, ≥5 years since last menses, and rigorously screened for coexisting disease. Patients were excluded for the following:1) Abnormality in any of the screening laboratory studies (CBC, serum calcium, phosphorus, albumin, alkaline phosphatase, creatinine, AST, 25-hydroxyvitamin D, and TSH); 2) Any fracture within the previous 6 months; 3) Presence of stage IV or V chronic kidney disease, chronic liver disease, severe neuropathic disease, unstable cardiovascular disease, malignancy, chronic gastrointestinal disease, hypo- or hyperparathyroidism, Cushing's syndrome, severe chronic obstructive pulmonary disease, alcoholism, or type 1 diabetes; 4) Undergoing treatment for blood clots, coagulation defects, or treatment with any of the following drugs: corticosteroids, anticonvulsant therapy, pharmacological doses of thyroid hormone, adrenal or anabolic steroids, aromatase inhibitors, calcitonin, calcium supplementation >1200 mg/d, bisphosphonates, estrogen, selective estrogen receptor modulators, parathyroid hormone, sodium fluoride, teriparatide, or TZDs. All subjects were required to have sufficient levels of vitamin D (serum 25-hydroxyvitamin D of >20 ng/ml).<br><br>For the second study, eligible participants were between 45 and 100 years old, diagnosed with osteoporosis and type 2 diabetes mellitus or prediabetes, and treated with DMab, oral or intravenous bisphosphonate, or calcium and vitamin D supplementation |





| | |
|---|---|
| | for at least 1 year. Subjects were excluded if baseline, or 6 or 12 month HbA1c, FPG and weight, and duration of type 2 diabetes mellitus or prediabetes were not available. |
| Recruitment | For the second study, all subjects were identified with the i2b2 search engine in the Mayo Clinic patient clinical data base, and their medical records were reviewed. |
| Ethics oversight | All clinical studies were approved by Mayo Clinic IRB. The samples used for in situ were obtained with approval by the Danish National Committee on Biomedical Research Ethics (S-20070121 and S-20120193). |

Note that full information on the approval of the study protocol must also be provided in the manuscript.

## Clinical data

Policy information about clinical studies

All manuscripts should comply with the ICMJE guidelines for publication of clinical research and a completed CONSORT checklist must be included with all submissions.

| | |
|---|---|
| Clinical trial registration | NCT02554695 |
| Study protocol | ClinicalTrials.gov |
| Data collection | The first clinical study was performed and samples collected in the outpatient Clinical Research and Trials Unit (CRTU) at the Mayo Clinic (Rochester, Minnesota, USA).<br><br>The second part of the study was a case control study of subjects with type 2 diabetes mellitus or prediabetes with osteoporosis treated at Mayo Clinic in Rochester, Minnesota, Scottsdale, Arizona, and Jacksonville, Florida, between January 1, 2009, and July 13, 2016. |
| Outcomes | For the first study (denosumab vs placebo), three months following the treatment, subjects returned to the CRTU; the baseline blood draw was repeated, and the subjects underwent sampling for bone marrow plasma and needle bone biopsies. Serum measures of bone metabolism were assessed compared to baseline, and bone biopsies were used for RNA isolation and gene expression analysis.<br><br>For the study of pre-diabetes and diabetes patients, outcomes of FPG, HbA1c, and weight were assessed at baseline, 6 months, and 12 months after subjects started osteoporosis treatment. The primary end point of this study was the change in HbA1c from baseline to 6 months and from baseline to 12 months after starting osteoporosis treatment. The secondary end points were the changes in FPG and body weight from baseline to 6 months and from baseline to 12 months after starting osteoporosis treatment. |
